# The *fliR* gene contributes to the virulence of *S. marcescens* in a *Drosophila* intestinal infection model

**DOI:** 10.1038/s41598-022-06780-w

**Published:** 2022-02-23

**Authors:** Bechara Sina Rahme, Matthieu Lestradet, Gisela Di Venanzio, Arshad Ayyaz, Miriam Wennida Yamba, Martina Lazzaro, Samuel Liégeois, Eleonora Garcia Véscovi, Dominique Ferrandon

**Affiliations:** 1 Université de Strasbourg, Strasbourg, France; 2 UPR 9022 du CNRS, Institut de Biologie Moléculaire du CNRS, CNRS, Strasbourg, France; 3 Instituto de Biología Molecular y Cellular de Rosario, Consejo Nacional de Investigaciones Cientificas y Tecnológicas, Universidad Nacional de Rosario, Rosario, Argentina; 4 Present Address: Department of Molecular Microbiology, Washington University School of Medicine, St Louis, MO 63110 USA; 5 Present Address: Department of Biological Sciences, University of Calgary, Calgary, Canada

**Keywords:** Bacterial infection, Mechanisms of disease, Pathogens, Bacterial host response, Bacterial pathogenesis, Intestinal diseases

## Abstract

*Serratia marcescens* is an opportunistic bacterium that infects a wide range of hosts including humans. It is a potent pathogen in a septic injury model of *Drosophila melanogaster* since a few bacteria directly injected in the body cavity kill the insect within a day. In contrast, flies do not succumb to ingested bacteria for days even though some bacteria cross the intestinal barrier into the hemolymph within hours. The mechanisms by which *S. marcescens* attacks enterocytes and damages the intestinal epithelium remain uncharacterized. To better understand intestinal infections, we performed a genetic screen for loss of virulence of ingested *S. marcescens* and identified FliR, a structural component of the flagellum, as a virulence factor. Next, we compared the virulence of two flagellum mutants *fliR* and *flhD* in two distinct *S. marcescens* strains. Both genes are required for *S. marcescens* to escape the gut lumen into the hemocoel, indicating that the flagellum plays an important role for the passage of bacteria through the intestinal barrier. Unexpectedly, *fliR* but not *flhD* is involved in *S. marcescens*-mediated damages of the intestinal epithelium that ultimately contribute to the demise of the host. Our results therefore suggest a flagellum-independent role for *fliR* in bacterial virulence.

## Introduction

Infectious diseases remain one of the leading causes of death in the world due to new emerging strains of pathogens, multidrug resistance of microorganisms, and persistent infections. The understanding of these diseases requires deep knowledge about the interactions between host and pathogen, which can be studied from two different perspectives: that from the host and that from the pathogen. Host defense against infections encompasses two distinct but complementary facets: resistance and resilience^1^, also referred to as disease tolerance^2–4^. Resistance, which relies on the immune system, is the ability of the host to directly attack the pathogen to lower the microbial burden and ultimately clear the infection. In contrast, resilience, is the ability of the host to withstand and repair damages provoked directly by the pathogen or indirectly by the host’s own immune response. Viewed from the pathogen perspective, the invading microorganism needs to survive inside the host, that is, to withstand, elude or neutralize host defenses, and to gather nutrients to sustain its growth, proliferation, and ultimately its dissemination.

The digestive tract is in constant contact with various pathogens that may occasionally contaminate the food. Therefore, the intestine has developed robust resistance and resilience mechanisms to confront and to endure such infections. Numerous reports have documented the use of *Drosophila* as a model to study intestinal infections^5–7^. It is thought that the fly midgut prevents the passage of bacteria to the hemolymph via three major arms^8^: the peritrophic matrix barrier, which is a passive physical defense mechanism, and two active chemical defense mechanisms, the local secretion in the lumen of Anti-Microbial Peptides (AMP) and the local release of Reactive Oxygen Species (ROS). The peritrophic matrix lines the gut epithelium and confines microorganisms to the lumen of the digestive tract^9,10^. The secretion of AMPs in the midgut is regulated by the IMD and not the Toll signaling pathway^10–13^. The detection of microbial uracil has been proposed to trigger the production of ROS in the lumen through the Dual Oxidase (DuOx) enzyme^14,15^. Additionally, at least three resilience mechanisms may contribute to maintain the homeostasis of the intestinal epithelium: (i) the secretion of Immune Response Catalase (IRC) limits the detrimental effect of ROS on intestinal epithelial cells (Enterocytes = ECs)^16^; (ii) the proliferation of Intestinal Stem Cells (ISCs) compensates EC cell death^17–20^; (iii) the extrusion of EC cytoplasm within hours of ingestion eliminates intracellular toxins and damaged organelles^21^. Even though the fly intestine harbors sophisticated defense mechanisms, some microorganisms, such as *Serratia marcescens* or *Pseudomonas aeruginosa,* are able to resist, survive, damage and cross the intestinal barrier^10,22,23^.

*S. marcescens* is a Gram-negative entomopathogen and also a human opportunistic bacterium associated with nosocomial infections^24–26^. The pathogenicity of *S. marcescens* relies on multiple virulence factors such as the pore-forming toxin hemolysin^27–29^, the serralysin protease^30–32^ or a phospholipase^33^. *S. marcescens* is a potent pathogen in the septic injury model of *Drosophila*. When introduced directly in the hemocoel, a few bacterial cells are sufficient to kill the fly within a day. The bacteria proliferate rapidly in the hemolymph causing bacteremia followed by death. Upon detection of the bacteria, the Immune deficiency (IMD) signaling pathway stimulates the secretion of AMPs by fat body cells. However, this systemic immune response does not affect *S. marcescens* since IMD-deficient flies are as susceptible as wild-type flies to septic injury^10^. However, in the oral infection model, *S. marcescens* invades and damages the intestinal epithelium and causes EC cell death; yet, the flies do not succumb to the infection for days. This delay is likely accounted by resilience mechanisms such as ISC compensatory proliferation^20^. In the midgut, the bacteria trigger the local release of AMPs by the IMD signaling pathway^10,12,13^ and are thought to induce the local secretion of ROS through the DuOx enzyme^15^. Interestingly, a low but significant number of bacteria can cross the intestinal barrier and manage to reach the hemolymph. In contrast to the septic injury model, *S. marcescens* is not able to proliferate in the hemocoel as it is controlled by phagocytosis. Indeed, phagocytosis-impaired flies are highly susceptible to the oral infection as ingested bacteria proliferate in the hemolymph^10^. In keeping with this cellular control of bacteria that have escaped in the hemocoel, the bacteria do not trigger the systemic immune response, which monitors short peptidoglycan fragments released by bacteria during their divisions^34^. Thus, under normal conditions, bacteria that have crossed the intestinal barrier do not appear to contribute to the virulence of this pathogen in the oral infection model.

The difference in the virulence of the bacteria between the septic injury and the oral infection model indicates that the virulence program of *S. marcescens* is downregulated after its passage from the gut lumen through the midgut epithelium to the hemolymph^10^. How *S. marcescens* modulates its virulence program according to its infection route remains unknown. Additionally, the virulence factors that the bacteria employ to damage the intestinal epithelium and to cross the fly intestinal barrier are still uncharacterized. To better understand intestinal infection by *S. marcescens,* we performed a small-scale genetic screen to isolate bacterial mutants displaying an impaired virulence in the *Drosophila* oral infection model. This screen identified a novel virulence factor, *fliR*, that is needed for *S. marcescens* to severely damage the intestinal epithelium and to efficiently kill the flies. Furthermore, this study sheds light on the importance of the flagellum for the dissemination of gut bacteria through the intestinal epithelium into the internal milieu potentially causing systemic infections.

## Results

### The *fliR* gene as a novel virulence factor in *S. marcescens*

To identify new virulence factors for *S. marcescens,* we partially screened a transposon (mini-Tn5) insertion mutant library generated in the Db10 strain^35^. We examined the survival of *eater*^−/−^ flies following the oral infection with individual mutant clones. Of note, the *eater*^−/−^ mutants are phagocytosis-impaired flies proven useful for the screen because of their susceptibility to wild-type *Serratia* intestinal infection: the bacteria proliferate in the hemolymph and rapidly kill the flies, making easier the selection for less virulent bacterial strains.

We have tested 1348 bacterial mutants and identified a strain (19H12) that exhibited reduced virulence in the intestinal infection (Supplementary Fig. 1). Sequencing analysis of the 19H12 clone revealed an insertion mutation in the gene *fliR,* which encodes a structural component of the flagellum and is required for its biosynthesis by participating in the export machinery of its components as well as some virulence factors^36–38^. FliR is a protein that forms part of the export gate of the flagellum, a structure embedded within the MS-ring, the basal body that anchors the flagellum to the cytoplasmic membrane and the cell wall.

### *fliR*, like *flhD*, is required for the formation of flagella in *S. marcescens*, as determined in in vivo studies

The function of *fliR* in the virulence of *Serratia* might be dependent on its role in the assembly of the flagellum. The latter is a complex process initiated by the major (class I) regulator FlhDC that controls the expression of several flagellar genes, including *fliR*^36^.

To validate the implication of *fliR* in the virulence of the bacteria, and to assess whether it is related to its function in the flagellum apparatus, we designed pKNOCK insertion mutants^39^ for the *fliR* gene as well as for the *flhD* regulatory gene. These insertion mutants, in addition to a *fliR* plasmidic rescue (*fliR* was cloned in the *pBB1:lacI:MCS* expression plasmid resulting in *pBB1:lacI:fliR*)^40^, were generated in two different *S. marcescens* wild-type strains of distinct origins: Db10 (a derivative of a *Drosophila* isolate from Stockholm, Sweden)^41^ and RM66262 (a clinical isolate from Rosario, Argentina)^42^.

After selecting mutants in both the Db10 and the RM66262 backgrounds, we first confirmed that the mutations in *fliR* or *flhD* do not alter the growth of the bacteria in the LB medium and in the infection solution (50 mM sucrose + 10% LB) (Supplementary Fig. 2). We then determined the loss of flagellum-dependent activities in all mutants (Supplementary Fig. 3): the flagellin expression is lost and the motility is impaired in the *flhD* and the *fliR* mutants as shown by western blot, swimming, and swarming assays. Also, the phospholipase of *S. marcescens* is secreted through the flagellum export system, which is a type 3 secretion system (T3SS)^38^. As expected, we did not detect phospholipase activity for either *flhD* or *fliR* mutants as compared to the wild-type strains (Supplementary Fig. 3). The flagellum-dependent functions of the bacteria are restored in the *fliR* rescue indicating that the observed phenotypes are due to the lack of *fliR* expression and that the two independent *fliR* mutations do not induce polar effects in the operon.

### *fliR* has a flagellum-independent role in the virulence of *S. marcescens* in the intestinal infection model

**Figure 1:**
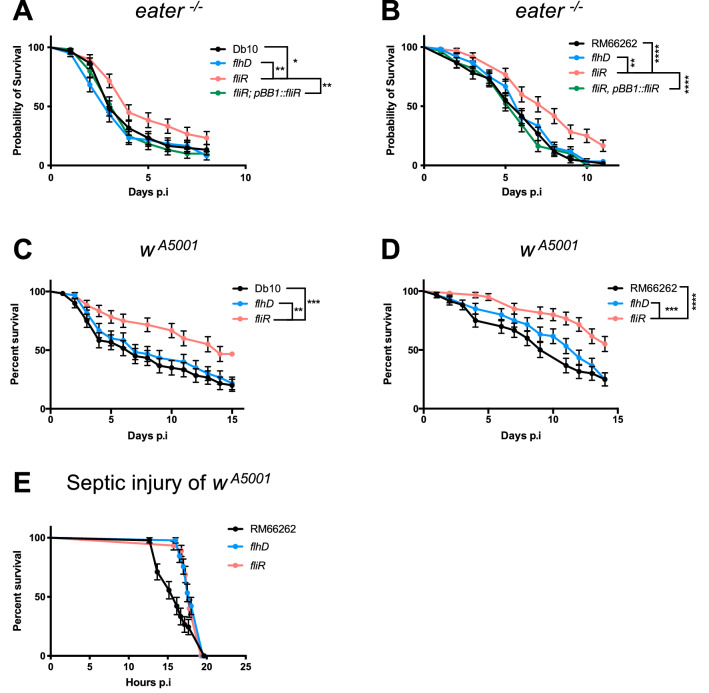
*fliR* and not *flhD* is required for *S. marcescens* full virulence in the *D. melanogaster* oral infection model. Flies were fed on filter pads with sucrose solution and 10% LB containing the bacteria at OD_600nm_ = 0.1. IPTG (0.5 mM) was added for the *fliR* rescue strain (**A**–**D**). (**A**, **B**) Survival test of *eater*^−/−^ mutant flies upon oral infection by bacterial strains in either the Db10 (**A**) or the RM66262 (**B**) genetic background. (**C**–**D**) Survival test of *w*^A5001^ flies upon oral infection by bacterial strains in either the Db10 (**C**) or the RM66262 (**D**) genetic background. (**E**) Survival test of *w*^A5001^ after injection of bacteria at OD_600nm_ = 0.1. The slightly faster lethality observed after a challenge with the wild-type strain was not confirmed in subsequent experiments. Each graph represents one out of three independent experiments that yielded similar results. Error bars represent the standard error. Statistical tests were performed using Log-rank.

Our first attempt in the in vivo study was to validate the result of the screen with *fliR* insertion mutant bacteria and to compare its virulence to the *flhD* bacteria in the oral infection model of phagocytosis-impaired flies. Indeed, *eater*^−/−^ flies fed with *fliR* mutant bacteria were less susceptible to the infection when compared to flies fed with either otherwise isogenic *flhD* mutant or wild-type bacteria of the Db10 (Fig. 1A) or the RM66262 (Fig. 1B) strains. Similar results were found when phagocytosis was impaired by the prior injection of latex beads that ultimately saturate the phagocytes after their engulfment (Supplementary Fig. 4A). As expected, the *fliR* strain harboring the plasmid rescue of *fliR* was as virulent as the wild-type strain, further demonstrating in vivo that the mutation in *fliR* is solely responsible for the observed phenotype (Fig. 1A,B). Additionally, we verified the virulence of the mutants in wild-type *w*^A5001^ flies. Likewise, *fliR* mutant bacteria in the two bacterial strain backgrounds were less virulent in the oral infection model when compared to the *flhD* mutant or wild-type bacteria (Fig. 1C,D). Moreover, we tested a possible role for *fliR* gene in the virulence of the bacteria in septic injury. We found that *fliR* mutant bacteria were as virulent as *flhD* mutant and wild-type *S. marcescens* when introduced directly in the hemolymph (Fig. 1E). In conclusion, these results reveal a *flhD*-independent role for *fliR* in the virulence of *S. marcescens* in intestinal infection, but not in the septic injury model. Similar results were obtained when monitoring the survival of IMD-deficient (*kenny*) and DuOx-deficient (silenced in ECs) flies following an oral infection (Supplementary Fig. 4B,C). These experiments indicate that the contribution of *fliR* in the virulence of *S. marcescens* is not related to its interactions with the fly immune system such as eliciting or evading the immune response during intestinal infections. As we had confirmed the role of *fliR* in the virulence of *S. marcescens* in two different bacterial strains, we focused only on the RM66262 strain for further investigations.

### The flagellum is essential for *S. marcescens* to traverse the epithelial barrier

**Figure 2:**
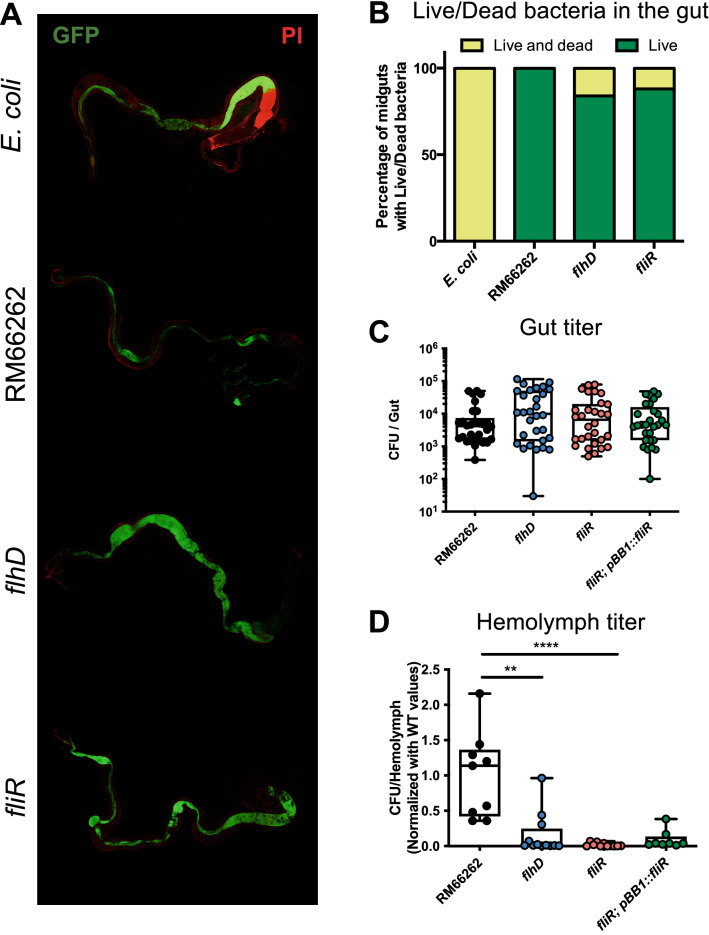
*flhD* and *fliR* mutants survive in the Drosophila midgut like wild-type *S. marcescens* but have decreased ability to cross the epithelial barrier. (**A**) Confocal pictures of *w*^A5001^ midguts after ingestion of GFP-expressing bacteria (green) and propidium iodide (PI) (red). Flies were fed on filter pads containing a mix of bacteria (OD_600nm_ = 10) and PI (50 µg/mL) for 4 h at 25 °C. (**B**) Quantification of *w*^A5001^ midguts with live or live and dead bacteria in the RM66262 background. Number of midguts per column = 10–12. (**C**) CFU count of bacteria in the midgut 24 h post-infection. *eater*^−/−^ mutant flies were fed with bacteria in the RM66262 background (OD_600nm_ = 0.1) at 25 °C. Each dot in the graph represent one infected midgut. Number of midguts per column = 30. (**D**) CFU count of bacteria in the hemolymph 4 h post-infection. *eater*^−/−^ mutant flies were orally infected by bacteria in the RM66262 background (OD_600nm_ = 0.1). Number of dots per column = 9–12. Statistical test was performed using Kruskal–Wallis and Dunn’s post-hoc tests (**C**, **D**). IPTG (0.5 mM) was added to the *fliR* rescue strain. Each graph represents one out of three independent experiments that yielded similar results, except for graph (**D**) that represent the pooled data of three independent experiments.

The bacteria in the gut lumen of flies are subjected to various stressors such as immune effectors and digestive enzymes. To monitor the survival of the *fliR* mutant in the digestive tract, we applied an assay consisting in the ingestion of bacteria that constitutively express GFP from a plasmid together with the propidium iodide stain: the GFP label indicates the presence of live bacteria, whereas the propidium iodide penetrates and stains only dead bacteria. We found that the intestinal lumen of *w*^A5001^ flies that ingested RM66262 wild-type, *flhD* or *fliR* mutants contained only live bacteria marked with GFP (Fig. 2A,B) as compared to the lumen of flies that have ingested the *E. coli* control. The absence of propidium iodide staining for the tested *S. marcescens* strains suggests that the mutants are not killed in the midgut at least at 4 h post-infection, whereas *E. coli* was killed in the posterior midgut after having passed through the acidic region (Fig. 2A,B). In addition, we measured the bacterial titer in the midgut of *eater* flies at 24 h post-infection. We observed that the CFU count of *fliR* mutants in the intestine is comparable to the values determined for either the *flhD* mutant or the wild-type bacteria (Fig. 2C). Taken together, these results indicate that *flhD* and *fliR* mutants are able to resist to the stressful environment of the midgut as well as wild-type bacteria.

Besides motility, adherence to and invasion of host cells are two other important functions of the flagellum^43,44^. Since flagellum mutants are not motile, we tested the ability of both *flhD* and *fliR* mutant bacteria to adhere to and invade CHO cells by forcing the contact between bacteria and host cells by centrifugation. As expected, we observed a decreased adhesion and invasion for both mutants in comparison to wild-type bacteria (Supplementary Fig. 5A,B). We also found that the invasion of *Drosophila* S2 cells by *fliR* bacteria is highly diminished when compared to wild-type bacteria (Db10) (Supplementary Fig. 5C). Therefore, these impaired functions of the flagellum may affect the ability of *S. marcescens* to traverse the intestinal epithelium and to cause septicemia. Therefore, to examine the ability of the bacterial mutants to cross the epithelial barrier, we quantified the amount of bacteria present in the hemolymph of phagocytosis-impaired flies 4 h after the beginning of RM66262 ingestion. We showed that both *flhD* and *fliR* bacteria were less abundant in the hemolymph as compared to wild-type bacteria (Fig. 2D). However, the ability of the *fliR* mutant to cross the intestinal barrier was not rescued by the complementation (Fig. 2D). Of note, the latter is carried out under the control of an inducible promoter that requires IPTG. The IPTG used to activate the expression of the *fliR* gene may have not been able to pass the intestinal barrier.

These results were confirmed upon dissection of wild-type midguts after gentamicin solution feeding to clear previously ingested bacteria remaining in the lumen. As gentamicin is not able to cross eukaryotic membranes, the microbial titer measured in the treated midguts corresponded to bacteria within ECs or adhering to the basal part of the epithelium, which is in contact with hemolymph^10^. We observed less *fliR* bacterial loads than wild-type ones (Supplementary Fig. 5D). In conclusion, both flagellum mutants exhibit difficulties to traverse the intestinal barrier. These findings pinpoint a requirement for the flagellum in the passage of the bacteria from the gut lumen to the body cavity.

### The *fliR *gene is required for *S. marcescens* to impact the homeostasis of the intestinal epithelium

**Figure 3:**
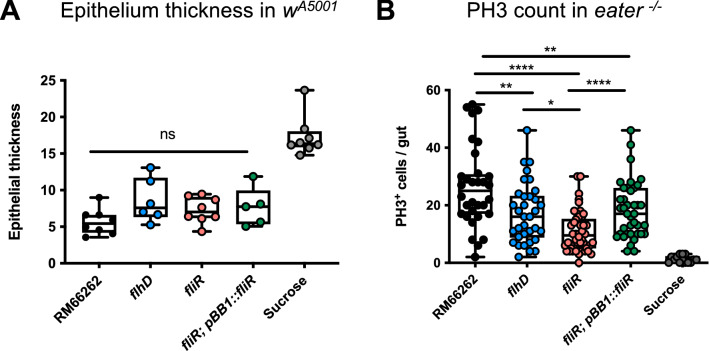
In comparison to *flhD*, *fliR* mutant bacteria trigger a lessened compensatory proliferation of *Drosophila* intestinal stem cells. (**A**) Epithelium thickness measured 3 h post-infection at 25 °C on *w*^A5001^ midguts stained with phalloidin (actin). The thickness was measured using the FIJI software, each dot represents the mean of 10 different measurements along the anterior midgut (R2 region). (**B**) Number of mitoses measured using a PH3 staining in whole midguts of *eater*^−/−^ mutant flies at 25 °C 24 h post-infection. Flies were orally infected with bacteria in the RM66262 background using OD_600nm_ = 10. IPTG (0.5 mM) was added in the infection solution containing the *fliR* rescue strain. Each graph represents three independent experiments. Statistical tests were performed using one-way ANOVA.

Following the ingestion of *S. marcescens*, two distinct resilience mechanisms are activated in the intestinal epithelium: the cytoplasmic purge and the compensatory proliferation of ISCs. In the early phase of infection, pore-forming toxins such as hemolysin elicit the extrusion of EC cytoplasm^21^. This short-term cytoplasmic purge prevents the toxic effect of the hemolysin on the ECs and results in a drastic thinning of the intestinal epithelium 3 h post-infection. We examined the induction of the cytoplasmic purge by measuring the thickness of the intestinal epithelium 3 h post-infection. The cytoplasmic purge was triggered in midguts infected with *fliR, flhD* mutants or the wild-type control, as the thinning of the epithelium (~ 10 µm) occurred in midguts infected with either mutant or control strains (Fig. 3A and Supplementary Fig. 6A). Thus, both mutants are toxic enough to trigger the cytoplasmic purge in ECs possibly because they secrete equivalent levels of hemolysin.

Despite several midgut defense mechanisms, the bacteria manage to inflict damages to the epithelium, to stress and to kill ECs via unknown virulence factors^10,20^. Subsequently, ISCs proliferate at 24 h in response to EC stress or death. A phosphohistone H3 (PH3) staining, which marks dividing ISCs in the gut, allows to indirectly monitor the extent of gut damages: an increase in the PH3 level results from an enhanced proliferation of ISC, which may reflect the extent of epithelial damage. Of note, ISC compensatory proliferation in response to EC cell death was previously detected throughout the midgut epithelium of flies that have ingested *S. marcescens*^20^. To examine the ability of both mutants to damage the intestinal epithelium, we performed a PH3 staining on *eater*^−/−^ infected midguts. We detected a significant decrease in the PH3-positive cell count in the midguts infected with *fliR* mutants as compared to the ones infected with either *flhD* mutants or wild-type RM66262 bacteria (Fig. 3B). Similar results were obtained following the infection of *w*^A5001^ flies with flagellar mutants in the Db10 genetic background (Supplementary Fig. 6B,C). This finding suggests a diminished efficiency for the *fliR* bacteria to attack the intestinal cells, a process which is at least partially independent from *flhD*.

## Discussion

Intestinal infection with *S. marcescens* shares similar features with *P. aeruginosa* oral infection including the passage through the epithelial barrier and the damages to ECs^10,22,23^. However, the mechanisms used by these two bacterial species to exert these two features has not yet been characterized. Here we have presented evidence that the flagellum of *S. marcescens* is required for its passage from the gut to the body cavity of the flies. Importantly, we have identified FliR as a novel virulence factor that is needed for the bacteria to severely damage the intestinal epithelium, apparently independently from its major function in building up flagella.

Bacteria can cross the intestinal barrier via two distinct strategies: paracellular/extracellular passage by swimming in between the closely apposed enterocytes through the septate junctions or intracellular passage through the intestinal cells. In this study, we showed that the flagellum of *S. marcescens* plays a crucial role in the passage of bacteria from the gut lumen to the hemolymph as both flagellar mutants *flhD* and *fliR* displayed decreased bacterial loads in the body cavity of the fly (Fig. 2D, Supplementary Fig. 5D). Most *S. marcescens* bacteria remain confined to the gut endoperitrophic compartment as the peritrophic matrix forms an efficient barrier^10^. It remains to be determined whether the flagellum is required for the passage through the peritrophic matrix of the few bacteria that manage to cross it. It has been previously shown that some bacteria were attempting to traverse the epithelium in between ECs at late stages of infection^10^. An open possibility is that for the early passage that occurs within 2 h of feeding, bacteria may cross at the proximal part of the midgut, in the proventriculus region where the peritrophic matrix is synthesized before being reinforced by ECs along the midgut^9,10^. In both cases, the role of the flagellum may be restricted to its motility function. *S. marcescens* appears to traverse the intestinal barrier more efficiently when they do not express hemolysin and therefore do not trigger the cytoplasmic purge enterocyte defense^21^. This observation suggests the possibility that *S. marcescens* crosses the epithelial barrier by invading intestinal cells, in keeping with a study that also showed that *S. marcescens* requires the flagellum to adhere to and invade CHO cells^44^ (Supplementary Fig. 5A,B). The lack of adherence and invasion observed for flagellar mutants can be related to the motility function of the flagellum or to the secretion, through the T3SS, of several virulence factors such as the phospholipase or the *S. marcescens* nuclease^38,45^. We note that in *Caulobacter crescentus*, the synthesis of the type IV pilus, which plays a primordial role in adherence, depends on flagellar genes for the production of pilin^46^.

Altogether, our results suggest that the escape of a few bacteria into the hemocoel does not contribute to the fatal outcome of the infection as the *flhD* mutant is as lethal as wild-type bacteria. This result is in keeping with the low bacterial burden detected in the hemolymph throughout the infection, which is limited by hemocytes that phagocytose *S. marcescens*^10^.

Here we showed that *fliR* mutants are less virulent in the intestinal infection model when compared to *flhD* mutant bacteria in two distinct *S. marcescens* strains (Fig. 1A–D). This difference in the virulence observed between *flhD* and *fliR* mutants implies a possible role for *fliR* in the virulence of the bacteria independently from its role in the flagellum or T3SS formation, which are both impaired in *fliR* and *flhD* mutants. A function of some, including a putative *fliR* homologue, but not all, flagellar genes in type IV pilus-dependent twitching motility has been reported in the nonflagellated bacterium *Lysobacter enzymogenes*, highlighting the possibility that some flagellar components have functions in bacterial physiology beyond the synthesis of the flagella^47^. Also, the *fliK* flagellar gene in *Bacillus thurengiensis* is required to counteract *Drosophila melanogaster* antimicrobial host defenses and is required for virulence independently of its role in the biosynthesis of the flagellum^48^.

**Figure 4:**
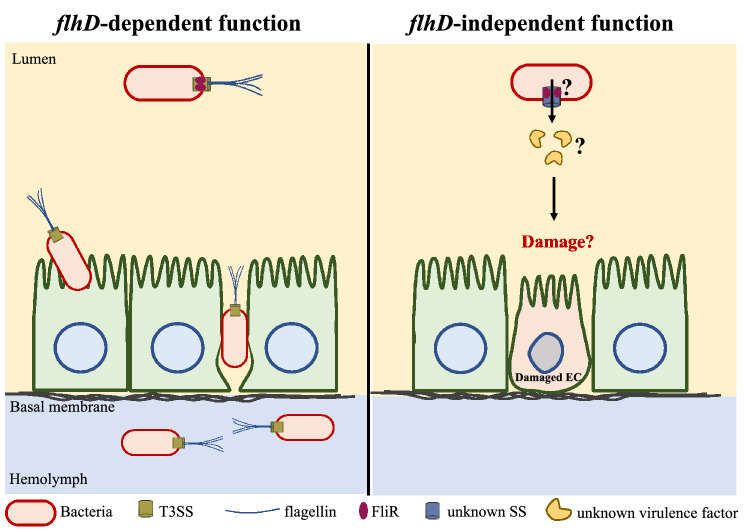
An *flhD-*independent function of *fliR* in the *Drosophila* intestinal infection model. (Left) The *flhD-*dependent function of *fliR* is restricted to its role in the flagellum formation. The flagellum is required for *S. marcescens* to traverse the epithelial barrier to reach the hemolymph. (Right) *fliR* acquired a function in the virulence of the bacteria independently from its role in the flagellum. *fliR* may be needed for the assembly of an *flhD-*independent secretory apparatus that would release virulence factors that may damage the intestinal cells.

On the one hand, the analysis of the epithelial thickness revealed a normal induction of the cytoplasmic purge following the infection with the *fliR* mutant bacteria (Fig. 3A and Supplementary Fig. 6A). This purge is triggered in response to the release of the hemolysin pore-forming toxin by *S. marcescens.* Therefore, the role of *fliR* in the virulence of the bacteria is not related to the secretion of hemolysin. Indeed, hemolysin is secreted by a T5SS^27,49,50^. On the other hand, we showed that the *fliR* strain is likely to induce less damage to the intestinal epithelium as the proliferation rate of ISCs is diminished in the midguts infected with *fliR* as compared to the ones infected with *flhD* bacteria (Fig. 3B and Supplementary Fig. 6B,C). This finding strongly suggests that *FliR* is implicated in the attack and the death of the ECs independently from its function in the flagellum. An attractive hypothesis is that FliR may be needed for the formation of a distinct secretory apparatus required for the secretion of unknown virulence factors that may directly attack and kill the fly intestinal cells (Fig. 4).

## Methods

### Fly strains

Flies were raised at 25 °C with 60% humidity on a semi-solid standard medium composed of 50 L of sterile water containing 3.2 kg of cornmeal, 2.4 kg of sugar, 580 g of yeast brewer’s dry powder, 240 g of agar and 260 g of 4-hydroxybenzoate sodium salt (Merck). The different fly strains used in the experiments were: *w*^A5001^ and *eater*^−/−^^51^.

### Bacterial strains and culture

Two strains of *S. marcescens* were used: Db10^41^ and RM66262^42^. The different mutants were generated by the pKNOCK plasmid insertion technique^39^. This plasmid carries antibiotic resistance to chloramphenicol (20 µg/mL) or to gentamicin (15 µg/mL). The *fliR; pBB1:lacI:fliR* (*fliR; pBB1::fliR*) strain expresses a wild-type copy of *fliR* under the control of an Isopropyl ß-D-1-thiogalactopyranoside (IPTG)-inducible promoter. The bacteria were cultured overnight on LB agar plates or in liquid medium at 37 °C with the corresponding antibiotics.

### Infection experiments and survival

The oral infection and the septic injury were performed at 25 °C essentially as described in Nehme et al.^10^. Bacterial pellet was diluted in 50 mM sucrose solution and 10% LB for the oral infection or in PBS for septic injury to a final OD_600_ of 0.1, 1 or 10 as needed. The survival of oral infected flies was monitored every day and 200 µL of 100 mM sucrose was added daily to the tubes.

### Bacterial loads

The bacterial titer of the intestine was measured 24 h post-infection. A single midgut was dissected and homogenized in 100 µL of PBS. The bacterial titer in the hemolymph was determined 4 h post-infection. The hemolymph was retrieved from five flies using a Nanoject II microinjector (Drummond) and collected in 10 µL of PBS. A serial dilution was applied on the samples, then each dilution was plated on LB-agar plates with ampicillin for the RM66262 wild-type strain and mutants thereof.

### Staining and imaging

To perform a propidium iodide staining, the flies were fed for 4 h with a solution containing 50 mM sucrose, 10% LB, bacteria that constitutively express GFP from a plasmid (OD_600_ of 10) and 50 µg/mL of propidium iodide. The midguts were dissected in PBS, fixed with 8% PFA then washed three times with PBS.

To measure epithelial thinning, midguts were dissected and fixed as described above. Actin staining was performed by incubating the samples for 1h30 in 10 µM of FITC-labeled phalloidin (Sigma-Aldrich #P5282). The epithelium thickness was measured using the FIJI software. The PH3 staining was performed at 24 h post-infection (OD_600_ of 10). The midguts were dissected in PBS, fixed with 8% PFA, incubated with the PH3 antibody (Millipore, ref 09-797) overnight at 4 °C, then stained with an anti-rabbit FITC-labeled antibody (Abcam #6717) overnight at 4 °C or 2 h at room temperature. All stained midguts were mounted in the Vectashield mounting medium (Vector Laboratories). The samples were observed and imaged using a LSM780 confocal microscope (Zeiss).

### Statistical analysis

All graphs and statistical tests were performed using GraphPad Prism. The statistical test used for the survival curves was Log-rank. Mann–Whitney, one-way ANOVA or Kruskal Wallis tests were performed for all other experiments (as specified in figure legends). The number of stars (*) represents the *P* values *P* ≥ 0.05 (ns), *P* < 0.05 (*), *P* < 0.01 (**), *P* < 0.001 (***) and *P* < 0.0001 (****).

## Supplementary Information

Supplementary Information.

## Data Availability

All data and materials are available upon request.
