# Supplementary material for: The fliR gene contributes to the virulence of S. marcescens in a Drosophila intestinal infection model
Source: Sci Rep. 2022 Feb 23;12:3068. doi: 10.1038/s41598-022-06780-w (PMC8866479; doi:10.1038/s41598-022-06780-w)
Supplement: Supplementary file 1 — Supplementary Information. [file 41598_2022_6780_MOESM1_ESM.pdf]

Supplementary Information to:

The *fliR* gene contributes to the virulence of *S. marcescens* in a *Drosophila* intestinal infection model

Bechara Sina Rahme[1], Matthieu Lestradet[1], Gisela Di Venanzio[2,3], Arshad Ayyaz[1,4], Miriam Wennida Yamba[1], Martina Lazzaro[2], Samuel Liégeois[1], Eleonora García Véscovi[2], Dominique Ferrandon[1*]

[1] Université de Strasbourg, CNRS, M3I UPR 9022 du CNRS, 67000 Strasbourg, France

[2] Instituto de Biología Molecular y Cellular de Rosario, Consejo Nacional de Investigaciones Cientificas y Tecnológicas, Universidad Nacional de Rosario, Rosario, Argentina

[3] Current location: Department of Molecular Microbiology, Washington University School of Medicine, St Louis, MO, 63110, USA.

[4] Current location: Department of Biological Sciences, University of Calgary, Canada

[*]Corresponding author: D.Ferrandon@ibmc-cnrs.unistra.fr

Supplementary methods

Supplementary fly stock

Fly strains used in the supplementary data are: w^{1118}; PBac(PB)*key*^{c02831} (*kenny* mutant) (Bloomington), *NP1-Gal4,Gal80*^{ts} [1,2] and *UAS-DuOx*^{RNAi} provided by Prof. Won Jae Lee. *NP1-Gal4,Gal80*^{ts} driver and *UAS-DuOx*^{RNAi} transgene were crossed at 18°C. The adult progeny was maintained at 29°C for 5 days prior to the experiment.

Latex beads injection

A volume of 69 nL of latex beads (Invitrogen) was injected in the hemolymph of *w*^{A5001} flies using the Nanoject II auto-nanoliter injector (Drummond).

Growth curves

Bacterial growth curves were performed in LB medium or in infection solution consisting of 10% LB and 50 mM sucrose. Overnight saturated bacterial cultures were diluted 1/100 in the appropriate solution. The growth was monitored at 37°C in a Synergy2 96-well microplate reader (BioTek). The OD_{600} was measured every hour for a period of 16h.

Western Blot anti-flagellin

Bacteria grown overnight were centrifuged at 5000 g for 10 min at 4ºC. The pellet was re-suspended in the sample solution: 120 mM Tris-HCl (pH 6.8), β-mercaptoethanol 0.5% (v/v), glycerol 2% (v/v), SDS 2% (w/v) and bromophenol blue 0.002% (w/v). The samples were heated for 5 min at 100ºC, then loaded into 12% (w/v) acrylamide gel. The electrophoresis was performed in 25 mM Tris buffer solution, 192 mM glycine (pH 8.3) and SDS 0.1% (w/v). The samples were then transferred to Hybond-ECL nitrocellulose membrane (GE Life Sciences) in

a semi-dry Trans-blot SD transfer tub (Bio-Rad). Subsequently, the membrane was incubated in TBS solution (20 mM Tris-HCl (pH 7.6), 137 mM NaCl) containing 5% (w/v) of skim milk powder. The membrane was further incubated for 1h with the primary polyclonal anti-fliC antibody, then washed three times with TBS and incubated for an 1h in the secondary polyclonal anti-rabbit antibody conjugated to enzyme alkaline phosphatase (Millipore) diluted in TBS (1:35000). Further, the membrane was washed then incubated with a solution containing the alkaline phosphatase substrates (BCIP 0.15 mg/mL and NBT 0.3 mg/mL) (Millipore).

Swimming and Swarming assays

The swimming assay was performed on 0.3% LB-agar plates. The plate was pricked with a toothpick carrying a bacterial colony. In contrast, the swarming assay was carried out on 0.6% LB-agar plate, on which 5 µL of an overnight culture was added. All plates were then incubated overnight at 30ºC.

Phospholipase activity

The assay was carried out on egg yolk plates. The yolk retrieved from the egg was washed then re-suspended in sterile PBS. The LB-agar was mixed with 5 mL of yolk. 5 µL of an overnight culture was added on the solid plates and incubated at 30°C overnight.

Adhesion and invasion assays in CHO cells

The Chinese Hamster Ovary (CHO) cells were cultured at 37°C with 5% CO_2 in minimum essential medium α-MEM supplemented with 10% of fetal bovine serum (Natocor), penicillin 10 mg/mL, amphotericin B 25 µg/mL and streptomycin 10 µg/mL. The pellet of overnight bacterial culture was washed then diluted in α-MEM to obtain a MOI of 2 then added to the cells. The plates were centrifuged at 1000xg for 10 min and were further incubated at 37°C for

1h. For the adhesion assay, the cells were washed with PBS solution containing 0.1% of triton and bacteria were retrieved then cultured on LB-agar plates overnight at 37°C. For the invasion assay, antibiotics (kanamycin 50 µg/mL and gentamicin 20 µg/mL) were added to the cells. The plates were then incubated for 120 or 360 min at 37°C before retrieving the bacteria from the cells. The bacteria were plated on LB-agar plates containing appropriate antibiotic and incubated overnight at 37°C.

Invasion assay in S2 *Drosophila* cells

The *Drosophila* S2 cell lines were maintained at 25°C in a Schneider (Biowest) medium supplemented with 10% of fetal bovine serum (SVF), 1% of GlutaMAX (Invitrogen), and 1% of PenStrep (Invitrogen). As the S2 cells are phagocytic and able to internalize the bacteria, the cells were treated with 10 µg/mL of cytochalasin D for 1h to block phagocytosis then washed with Schneider medium. The pellet of overnight bacterial culture was washed then diluted in Schneider medium to obtain a MOI of 5 or 10 then added to the cells for 1h. Further, the S2 cells were treated with gentamicin (100 µg/mL) for 1h, washed with Schneider medium, then lysed by sonification for 15 min and subsequent centrifugation at 4000 rpm. The retrieved bacteria were plated on LB-agar plate containing appropriate antibiotic and incubated overnight at 37°C.

Gentamicin treatment

One day following an oral infection, the flies were fed on a solution containing PBS and gentamicin (100 µg/mL) for 2h. After, the flies were shifted to a tube containing a sucrose solution (100 mM) to wash away the antibiotics for 30 min and the procedure was repeated twice. The dissected midguts were dipped in 70% ethanol, crushed in sterile PBS then plated on LB-agar plates containing streptomycin (100 µg/mL).

Bibliography

1. Nehme, N. T. *et al.* A model of bacterial intestinal infections in Drosophila melanogaster. *PLoS Pathog.* **3**, 1694–1709 (2007).

2. Cronin, S. J. *et al.* Genome-wide RNAi screen identifies genes involved in intestinal pathogenic bacterial infection. *Science.* **325**, 340–343 (2009).

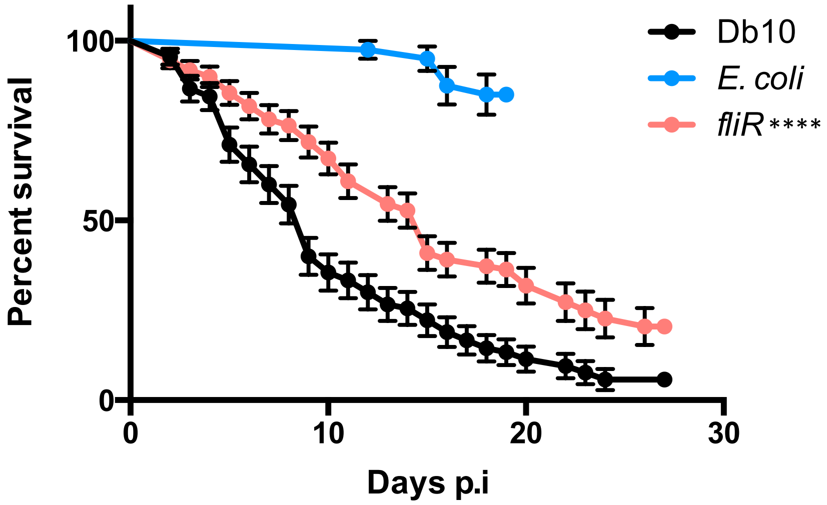

Supplementary Fig. 1: The Db10 19H12 *fliR* mutant clone is less virulent than WT bacteria in the intestinal infection model

Survival test of *eater*$^{-/-}$ mutant flies to the initial miniTn5 transposon insertion mutant of the *fliR* gene picked up in the genetic screen (OD_{600nm} = 1). Error bars represent the standard error. Statistical test was performed using Logrank.

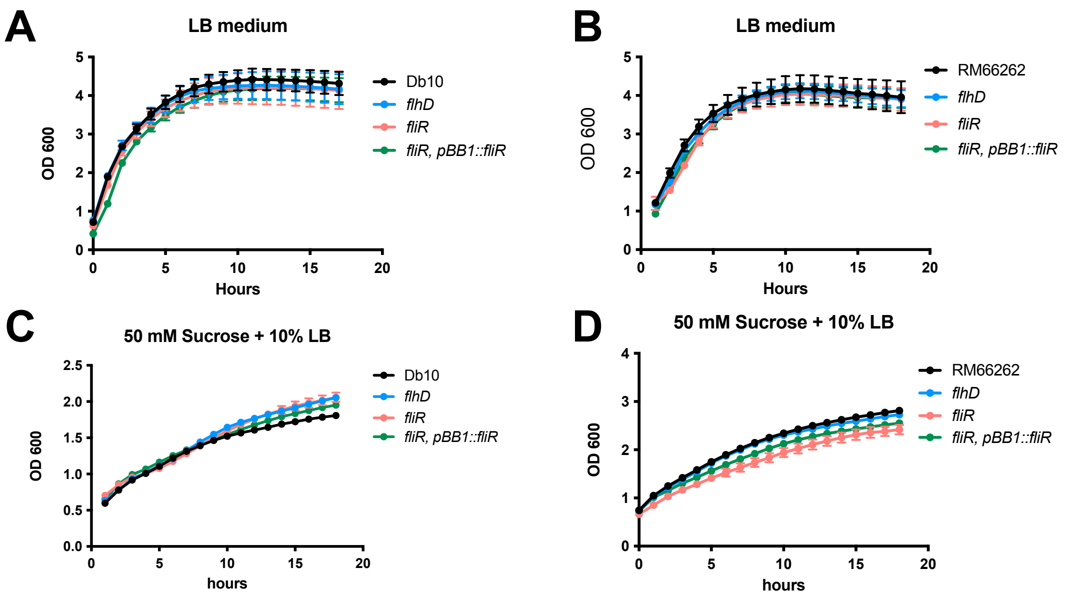

Supplementary Fig. 2: *fliR* and *flhD* mutant bacteria display a normal growth in LB and in infection solution

(A-B) Growth curve in LB medium for RM66262 (A) and Db10 (B) candidates. **(C-D)** Growth curve in infection solution containing 50 mM sucrose and 10% LB for RM66262 (C) and Db10 (D) candidates. OD_{600nm} was measured each hour at 37° C. Error bars represent the standard error of the mean (SEM). Each graph represents one out of three independent experiments that yielded similar results.

A

	WT strain (RM66262 or Db10)	*flhD*	*fliR*	*fliR; pBB1::fliR*
Growth in LB	+	+	+	+
Growth in infection solution	+	+	+	+
Flagellin	+	-	-	+
Swimming	+	-	-	+
Swarming	+	-	-	+
Phospholipase activity	+	-	-	+

B **C** **D** **E** **F**

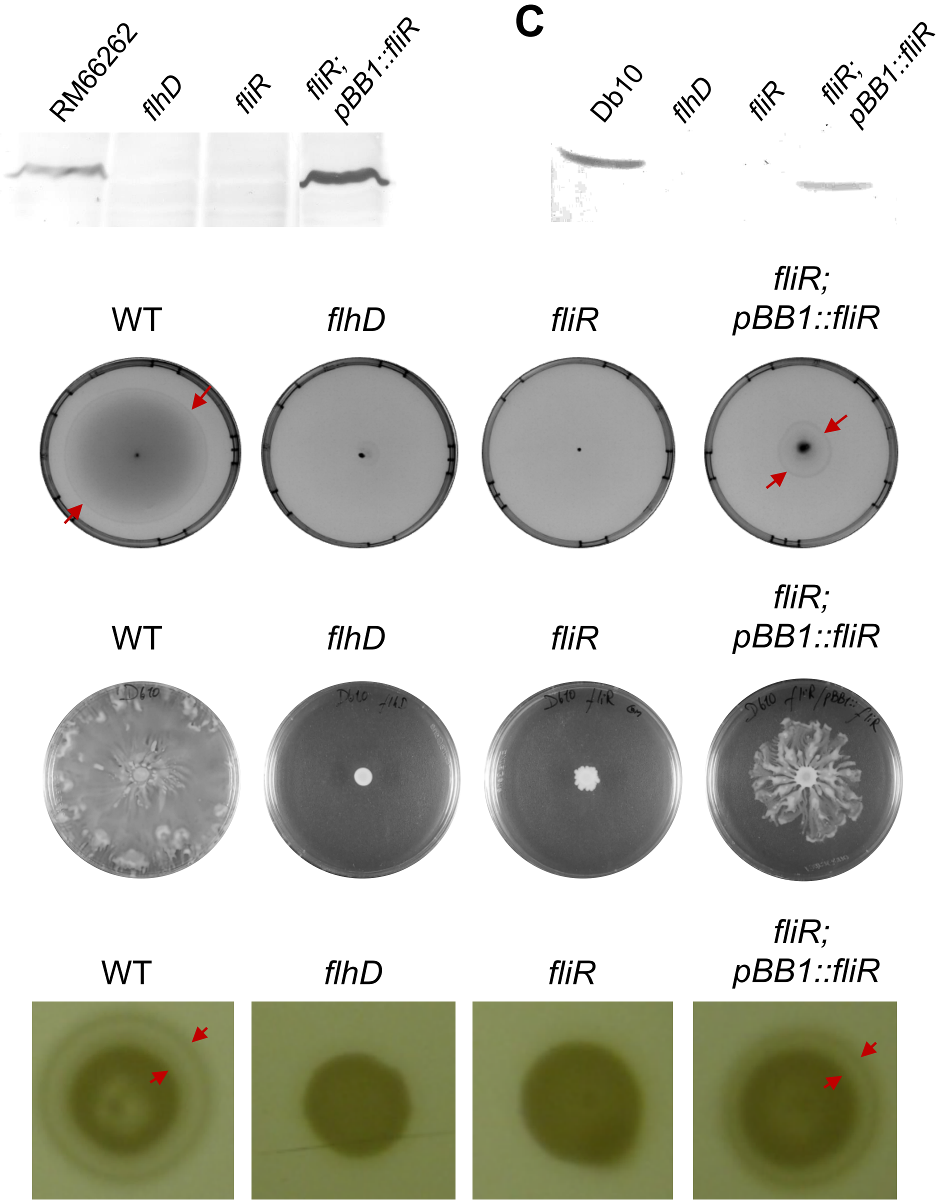

Supplementary Fig. 3

Supplementary Fig. 3: Impaired motility and flagellum-dependent secretion for *flhD* and *fliR* mutants

(**A**) Table displaying the results of different *in vitro* assays comparing *flhD* and *fliR* mutants to wild-type bacteria in the two different genetic backgrounds, RM66262 and Db10. **(B-C)** Western blot using an anti-flagellin antibody on RM66262 (B) and Db10 (C) strains. **(D)** Swimming assay on 0.3% LB-agar plate at 30° C. Red arrows delimit the halo. **(E)** Swarming assay on 0.6% LB-agar plate at 30° C. **(F)** Test for phospholipase activity on egg yolk plate at 30° C. Red arrows delimit the halo. D-F: pictures representative of experiments performed on the Db10 and RM66262 strains. In all experiments, 0.5 mM IPTG was added for the *fliR* rescue strain. Bacterial strains were grown at 30° C for 16 hours.

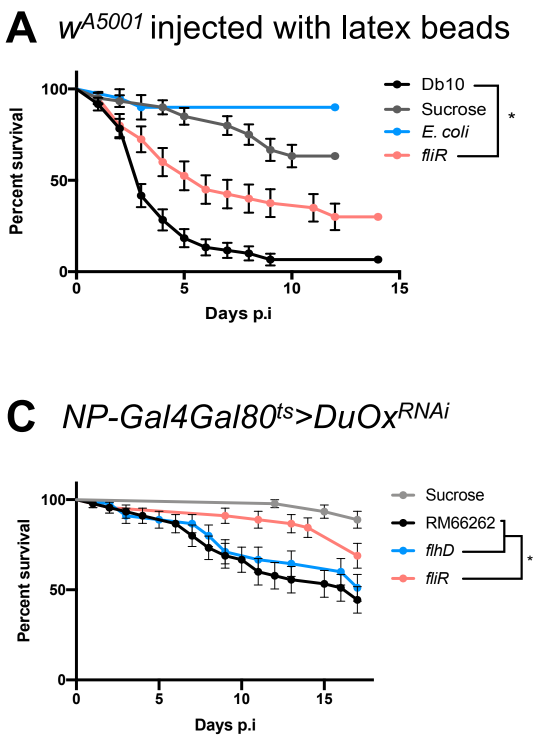

Supplementary Fig. 4: *fliR* mutant bacteria are less virulent compared to wild-type bacteria in the oral infection model on flies deficient for host defenses

(A) Survival test of w^{A5001} flies injected with latex beads (phagocytosis-impaired). Flies were fed at 25ºC on filter pads containing the bacteria (OD_{600nm} = 1). **(B)** Survival test of *kenny* mutant flies (IMD-pathway deficient). The infection was done at 25ºC using OD_{600nm} = 0.1. **(C)** Survival test of *NP1-Gal4Gal80^{ts}>UAS-DuOx^{RNAi}* flies at 29°C. Flies crossed at 18°C were kept at 29°C for 5 days prior to the infection to activate the DuOx^{RNAi}. Error bars represent the standard error. Logrank statistical test was used. 200 µL of sucrose (100 mM) were added every day.

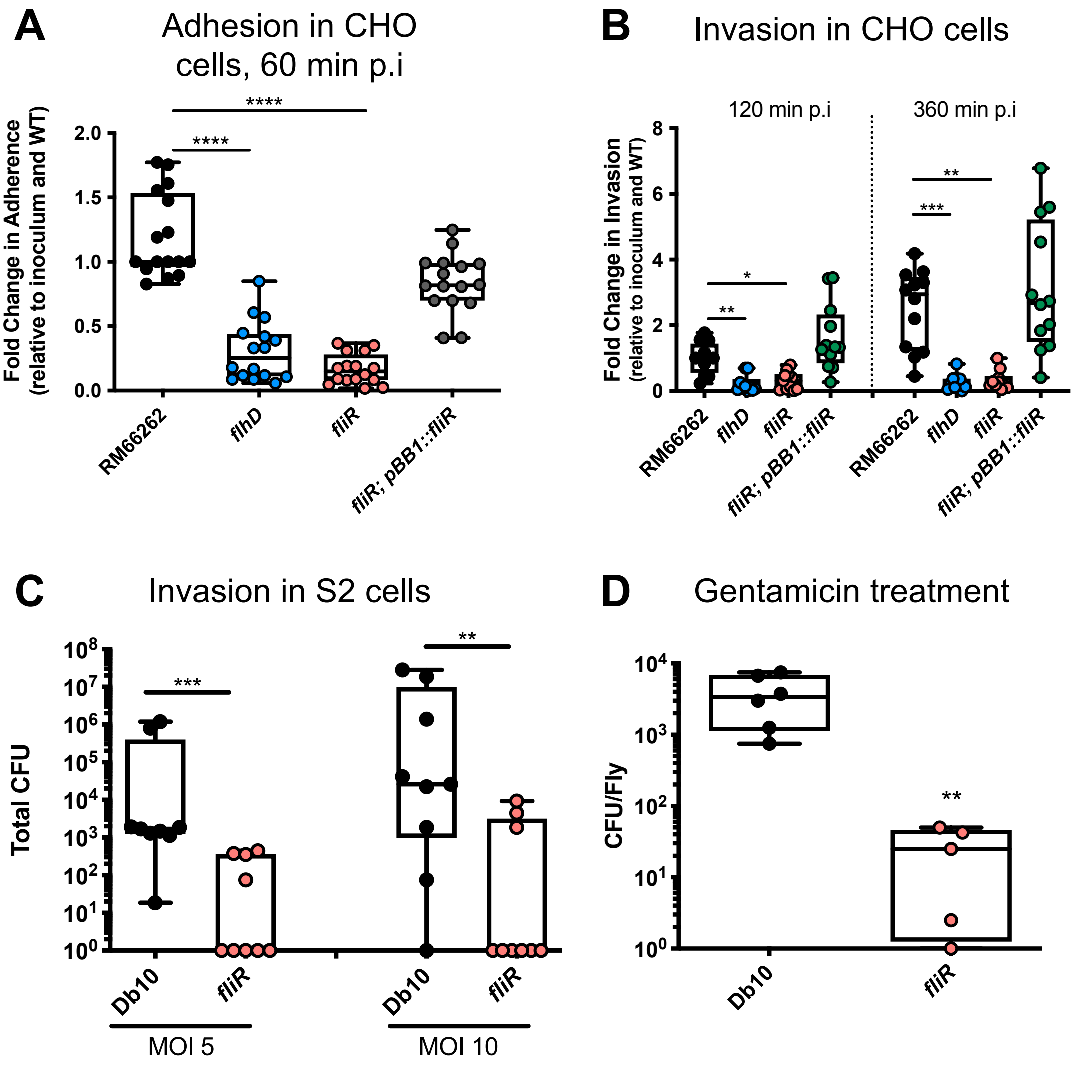

Supplementary Fig. 5: The ability of *flhD* and *fliR* mutants to adhere and invade mammalian or Drosophila cultured cells is impaired

(A) Adhesion assay of RM66262 candidates to CHO cells. The number of adherent bacteria was counted after 60 min of infection. **(B)** Invasion assay of RM66262 candidates to CHO cells. The number of intracellular bacteria was counted 120 min and 360 min after infection. Cells were incubated at 37°C with bacteria-contaminated αMEM medium with MOI=2. IPTG (0.5 mM) was added to the medium containing the rescue strain (n=4) (A-B). **(C)** Invasion assay of Db10 and *fliR* mutant in S2 *Drosophila* cells pretreated with cytochalasin D. The cells were incubated at 37ºC with bacteria-containing Schneider medium with MOI=5 or 10. **(D)** Bacterial count in the intestinal epithelium. After the infection, the flies were fed with gentamicin (100 µg/mL) solution then fed with sterile sucrose. The CFU count of crushed flies represents the number of bacteria inside enterocytes or adhering to the basal side of the intestine. Each graph represents one out of three independent experiments that yielded similar results. Statistical tests were performed using Kruskal-Wallis and Dunn's post-hoc tests (A-B) and Mann-Whitney (C-D).

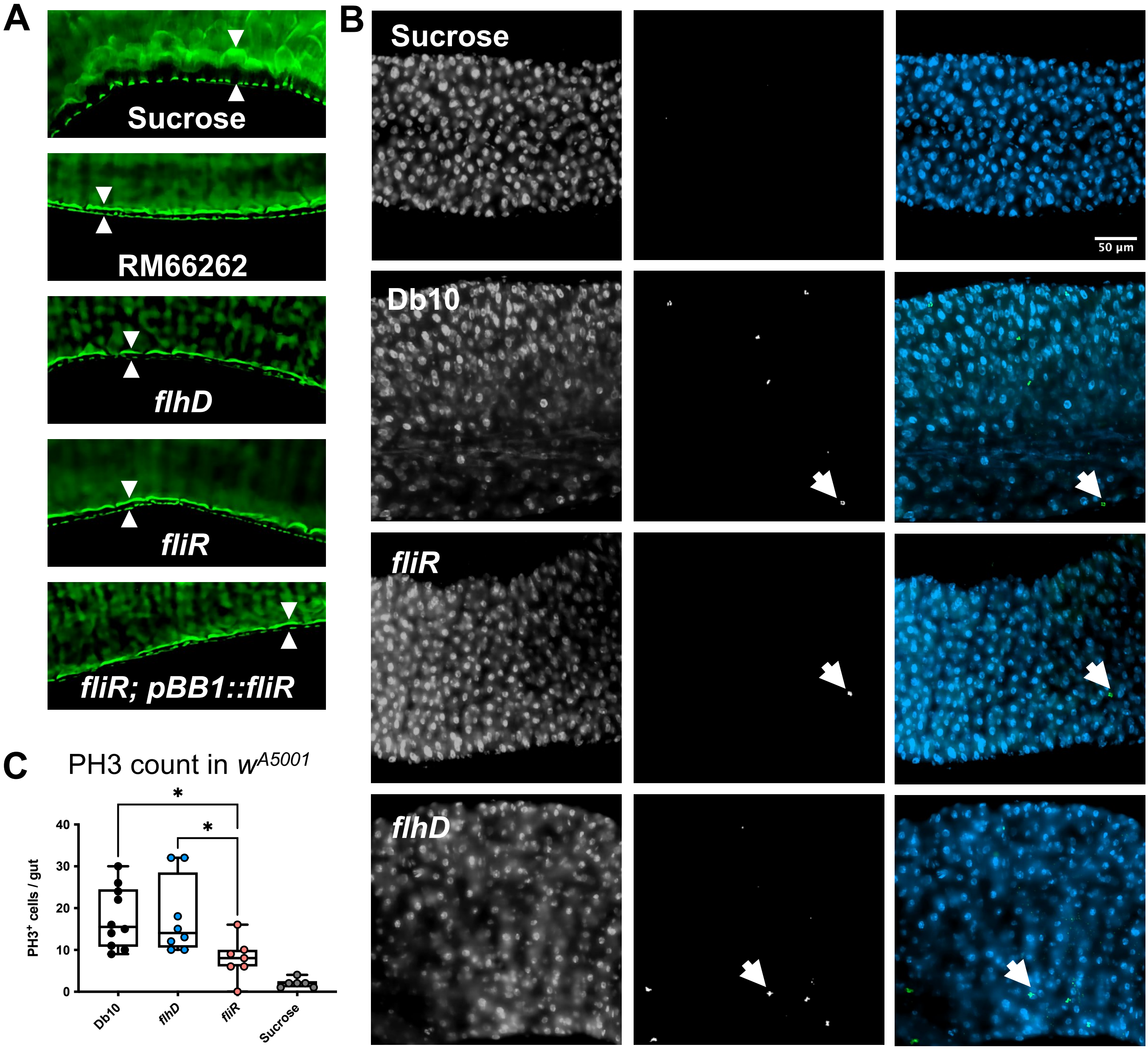

Supplementary Fig. 6: The compensatory proliferation of *Drosophila* stem cells is decreased in response to *fliR* and not to *flhD* infection

(A) Microscopy pictures of a segment of the intestinal epithelium (R2 region) of w^{A5001} flies stained with phalloidin (green). White arrows delimit the epithelial thickness. Flies were orally infected with RM66262 candidates using OD_{600nm} = 10 for 3 hours. **(B)** Microscopy pictures of the R4 region of w^{A5001} midguts stained with PH3 antibody (Green) and DAPI (Blue). White arrows indicate proliferating intestinal stem cells. **(C)** Quantification of mitoses measured using PH3 staining in whole midguts of w^{A5001} flies. Statistical test performed using One-way ANOVA. (B-C) Flies were orally infected with the Db10 strain using OD_{600nm} = 10 at 25° C for 24h.
